# The Latest of the Water Cures

**Published:** 1887-02-05

**Authors:** 


### The Latest of the Water Cures.

Since the days when the late Bulwer Lytton wrote "Life at the Water Cure," the science of Therapeutics has passed through phases of almost infinite variety. Great are the virtues of water! Whether cold or hot; whether used internally or externally, the fluid which costs the world nothing for production has properties of the rarest kind, and produces effects of the first order of importance. Our business now is with water taken internally; and with a peculiar kind of water—viz., *Mineral Water*, or water plus a variety of salts, which make of it something quite different from the simple combination of hydrogen and oxygen to which we are wont to apply the familiar name "water." Mineral Waters are very fashionable at the present time, and deservedly so. Few things are better suited to the condition induced by an over-worked stomach, and a too much burdened liver than those waters which contain in happy proportions certain dissolved salts of well-known potency in stomach and liver derangements. Blue Pill! We will not revile him! He is an old and well tried friend, and cannot be sent packing at the bidding of any of your new and fashionable Podophyllins or Euonymins, or even in the interests of the more pleasant and hardly less efficacious mineral waters. But still he is a friend who is not always perfectly agreeable. Too great an intimacy with him is apt to produce consequences which are, to say the least, unpleasant, and sometimes it must be confessed, decidedly injurious. If, therefore, we can find a substitute which in all ordinary cases will answer our purpose quite as well, we are not disinclined to give it a very friendly welcome. Such a substitute is to be found in certain mineral waters. It must be borne in mind, however, that there are waters and waters, and that which is good for one person may be very unsuitable for another. Therefore, some caution is required in the use of these favourite remedies. It is said, with what truth we do not know, that the following quaint epitaph may be seen in a certain country churchyard.

"Here lies I and my two daughters.
Who died from drinking the Cheltenham waters
If we had kept to the Epsom salts
We shouldn't have been in these here vaults."

Clearly, therefore, it is necessary to exercise some discrimination in the choice of these apparently harmless fluids, at any rate if the voice from the "vaults" is allowed any warning weight. As a rule the best method of finding out the most suitable water is to try several kinds, to try them repeatedly, and under a variety of circumstances. In ordinary conditions of health this is generally a perfectly safe course to pursue, few of the waters being strong enough to do any serious harm. But if there be any uncertainty as to the state of the health, it will be well to take counsel of the family physioian. We say the *family physician*, advisedly, because in most cases he is the leech who best knows the constitution, and is therefore most to be trusted.

It has been our fortune lately to experiment in various ways with *Æsculap water*. From its composition, and especially from its richness in the sulphates of soda and magnesia, it ought theoretically to be of great service as a gentle but effective saline. Experience has shown that such an expectation is justified by results. It is an excellent water both for occasional and general use, and has the somewhat rare property of being capable of manipulation so as to form a really pleasant drink. For this purpose take half a tumbler of the water, the juice of half a lemon, two or three pieces of sugar: fill up with boiling water, and drink the mixture hot or cold. It is quite safe, and may be used by ladies and children, in appropriate doses, as well as by members of the stronger sex. It has already gained for itself a high place among the articles of its class, and is likely, with growing use, to attain an increased and well-deserved popularity. The *mineral water cure* is probably as well worthy of the consideration of chronic dyspeptics and others as the famed water cure which is associated with the name of Priessnitz, and to promote the advancement of which such magnificent temples have been reared in our own and other countries.

